# Food stress causes sex-specific maternal effects in mites

**DOI:** 10.1242/jeb.123752

**Published:** 2015-08

**Authors:** Andreas Walzer, Peter Schausberger

**Affiliations:** Arthropod Ecology and Behavior Group, Division of Plant Protection, Department of Crop Sciences, University of Natural Resources and Life Sciences, Peter Jordanstrasse 82, Vienna 1190, Austria

**Keywords:** Egg size/number trade-off, Environmental stress, Trans-generational effects, Sex-specific effects, Phytoseiid mites

## Abstract

Life history theory predicts that females should produce few large eggs under food stress and many small eggs when food is abundant. We tested this prediction in three female-biased size-dimorphic predatory mites feeding on herbivorous spider mite prey: *Phytoseiulus persimilis*, a specialized spider mite predator; *Neoseiulus californicus*, a generalist preferring spider mites; *Amblyseius andersoni*, a broad diet generalist. Irrespective of predator species and offspring sex, most females laid only one small egg under severe food stress. Irrespective of predator species, the number of female but not male eggs decreased with increasing maternal food stress. This sex-specific effect was probably due to the higher production costs of large female than small male eggs. The complexity of the response to the varying availability of spider mite prey correlated with the predators' degree of adaptation to this prey. Most *A. andersoni* females did not oviposit under severe food stress, whereas *N. californicus* and *P. persimilis* did oviposit. Under moderate food stress, only *P. persimilis* increased its investment per offspring, at the expense of egg number, and produced few large female eggs. When prey was abundant, *P. persimilis* decreased the female egg sizes at the expense of increased egg numbers, resulting in a sex-specific egg size/number trade-off. Maternal effects manifested only in *N. californicus* and *P. persimilis*. Small egg size correlated with the body size of daughters but not sons. Overall, our study provides a key example of sex-specific maternal effects, i.e. food stress during egg production more strongly affects the sex of the large than the small offspring.

## Abstract (precis)

**Summary:** The predatory mite *Phytoseiulus persimilis* produces few large female eggs under moderate food stress but many small eggs when food is abundant, suggesting a sex-specific trade-off exists between egg size and number.

## INTRODUCTION

How should a female cope with food limitation during the reproductive phase? Life history theories predict that females should flexibly respond to food limitation and adjust their reproduction to the prevailing environmental conditions to optimize their own and offspring fitness (Roff, 1992; Stearns, 1992). Two prime traits modified by females under food stress are offspring number and size (Roff, 1992; Stearns, 1992; Fox and Czesak, 2000). Food-stressed females commonly shift energy from current reproduction to their own survival and future reproduction by reducing offspring number (for insects: Gwynne and Brown, 1994; Agarwala et al., 2008; gastropods: Spight and Emlen, 1976; reptiles: Andren and Nilson, 1983; birds: Hussell and Quinney, 1987; mammals: Duquette and Millar, 1995) and/or by reducing offspring size in favor of offspring number (Fox and Czesak, 2000; Bonduriansky and Head, 2007). In size-dimorphic species, food-stressed females may additionally, or alternatively, adjust offspring sex ratio because of differing production costs of sons and daughters (Trivers and Willard, 1973; Charnov, 1982). Maternal adjustment of offspring size may have profound effects on both maternal and offspring fitness, independent of any genotypic effects (Mousseau and Fox, 1998; Bonduriansky and Day, 2009). Maternal or trans-generational life history effects triggered by food stress during the reproductive phase may influence offspring survival, growth, developmental time and/or body size (Bashey, 2006; Johnson et al., 2014). Moreover, in size-dimorphic species, maternal effects may only affect one but not the other sex (Macke et al., 2011). For example, food-stressed females could only reduce the egg size of the larger sex, resulting in sex-specific phenotypic changes of offspring body size at maturity.

Here, we investigated the effects of maternal food stress on fitness-relevant maternal and offspring life history traits in the plant-inhabiting predatory mites *Phytoseiulus persimilis*, *Neoseiulus californicus* and *Amblyseius andersoni* (Acari: Phytoseiidae). These three species co-occur in the Mediterranean region sharing spider mites of the genus *Tetranychus* as prey (De Moraes et al., 2004; Walzer and Schausberger, 2011a). Spider mites are an ephemeral prey because of their rapid successive phases of host plant colonization, population growth, dispersal and local extinction (Sabelis and Dicke, 1985). Their predators thus frequently experience food stress during the juvenile and reproductive phases. However, the three predators differ in their adaptations to exploit spider mite prey: *P. persimilis* is highly specialized on spider mites; *N. californicus* prefers spider mites but can also use other animal and non-animal food; *A. andersoni* is a broad diet generalist without any known preference (McMurtry and Croft, 1997). Accordingly, we assumed that the more specialized a mite is on spider mite prey, the more likely they are to evolve sophisticated strategies to cope with food stress.

The reproductive output of all three species correlates with prey availability (Amano and Chant, 1978; Friese and Gilstrap, 1982; Vanas et al., 2006), characterizing them as typical income breeders (Stephens et al., 2009). Gravid females disrupt egg production under food limitation and reproduce again, without re-mating, when conditions improve (Sabelis, 1985). Simple maternal care is evident from selective egg placement based on food availability and offspring predation risk (Vanas et al., 2006; Walzer et al., 2006; Schausberger and Hoffmann, 2008; Walzer and Schausberger, 2011b, 2012). Phytoseiid mites are pseudo-arrhenotokous, i.e. both male and female eggs are fertilized and diploid at the beginning. However, the paternal genome disappears during egg development, resulting in haploid males (Sabelis et al., 2002). Males are smaller than females in all three species (Walzer and Schausberger, 2011a). Size dimorphism is already apparent in the egg stage of *P. persimilis* (Sabelis et al., 2002), but has not yet been assessed in *N. californicus* and *A. andersoni*. All three species have a maternally controlled female-biased sex ratio (Sabelis et al., 2002), ranging from 0.6 to 0.8 under optimal environmental conditions (Amano and Chant, 1978; Walzer and Schausberger, 2014).

We determined the effects of maternal food stress – limited spider mite availability – on the sex-specific offspring number and size of *P. persimilis*, *N. californicus* and *A. andersoni* in the lab. Based on life history theories (Roff, 1992; Stearns, 1992), we hypothesized that: (1) maternal food stress more strongly affects female than male egg number and size; (2) maternal effects are sex-specific, i.e. maternal food stress more strongly affects the larger daughters than small sons; and (3) stronger adaptation on spider mite prey correlates with more sophisticated maternal adjustment strategies to cope with food stress.

## RESULTS

### Oviposition

**Fig. 1.**
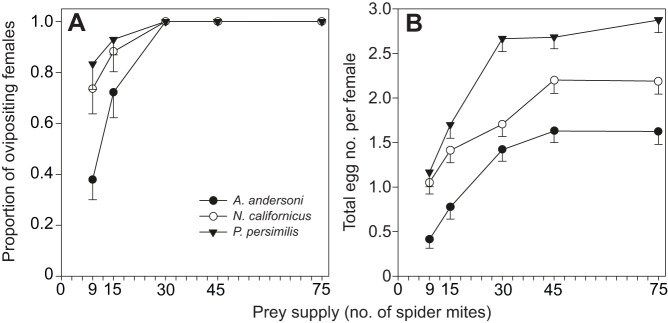
**Oviposition under food stress in mites.** Oviposition probability (A) and egg production (B) (means–s.e.m.) of *Phytoseiulus persimilis*, *Neoseiulus californicus* and *Amblyseius andersoni* females as a function of maternal prey supply (spider mite eggs and juveniles at 2:1 ratio).

Female body size did not affect the oviposition probability at a prey supply of 9 (GLM; Wald χ^2^_1_=1.478, *P*=0.224) and 15 spider mites (Wald χ^2^_1_=1.115, *P*=0.291); however, the factor species influenced the oviposition probability at 9 spider mites (Wald χ^2^_2_=9.555, *P*=0.008), but not 15 spider mites (Wald χ^2^_2_=2.749, *P*=0.253). When provided with 9 spider mites, significantly fewer *A. andersoni* females produced eggs compared with *P. persimilis* and *N. californicus* females (pairwise LSD tests, *A. andersoni* versus *N. californicus*: *P*=0.007; *A. andersoni* versus *P. persimilis*: *P*=0.001; *N. californicus* versus *P. persimilis*: *P*=0.513) (Fig. 1A). The total oviposition (number of deposited eggs) differed among species (Wald χ^2^_2_=70.506, *P*<0.001) and prey densities (Wald χ^2^_4_=217.417, *P*<0.001). The interaction of species and prey density (Wald χ^2^_8_=9.091, *P*=0.335) and maternal body size (Wald χ^2^_1_=0.620, *P*=0.431) did not have an effect. Across prey densities, *A. andersoni* laid fewer eggs (1.20±0.06; mean±s.e.m.) than *N. californicus* (1.73±0.07) and *P. persimilis* (2.20±0.10) (*P*<0.001 for all pairwise LSD tests). Across species, egg number increased with prey density (*P*<0.05 for all pairwise comparisons) except between 45 and 75 spider mites (Fig. 1B).

### Sex-specific egg number

**Fig. 2.**
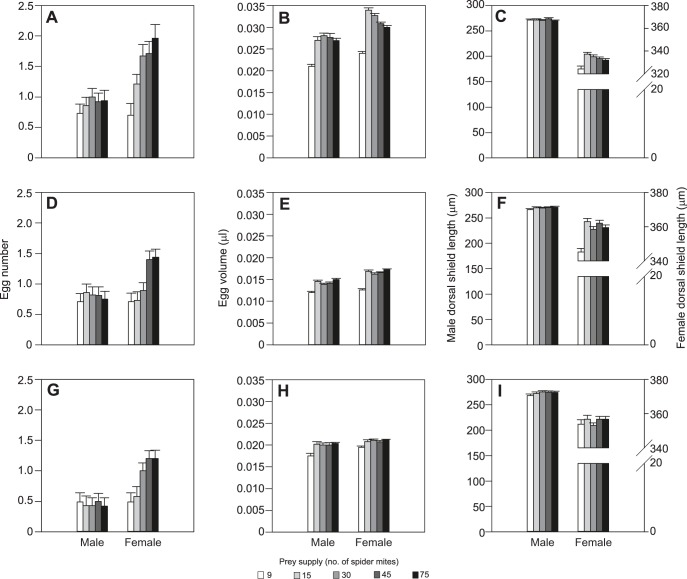
**Effects of maternal food stress on sex-specific egg number, size and offspring size.** Male and female egg numbers (A,D,G), egg volumes (B,E,H) and offspring sizes at maturity (C,F,I) produced by *P. persimilis* (A–C), *N. californicus* (D–F) and *A. andersoni* (G–I) females (mean+s.e.m.) in dependence on prey supply levels.

Maternal body size did not affect the number of eggs laid by *P. persimilis*, *N. californicus* and *A. andersoni*. In each species, both prey density and offspring sex as main factors, and their interaction, affected the number of eggs laid (Tables 1–3). The females of all three species produced similar numbers of female and male eggs at 9 spider mites (pairwise LSD tests, *P. persimilis*: *P*=1.000; *N. californicus*: *P*=1.000; *A. andersoni*: *P*=0.847). Higher prey densities increased the number of female (*P. persimilis*, 9 versus 15 spider mites: *P*=0.014, 15 versus 45 spider mites: *P*<0.001; *N. californicus*, 9, 15, 30 versus 45 and 75 spider mites: *P*<0.01; *A. andersoni*, 9 and 15 versus 45 spider mites: *P*<0.010) but not male eggs (*P. persimilis*: *P*>0.127 for all pairwise comparisons; *N. californicus*: *P*>0.457; *A. andersoni*: *P*>0.574) (Fig. 2A,D,G).

**Table 1.**
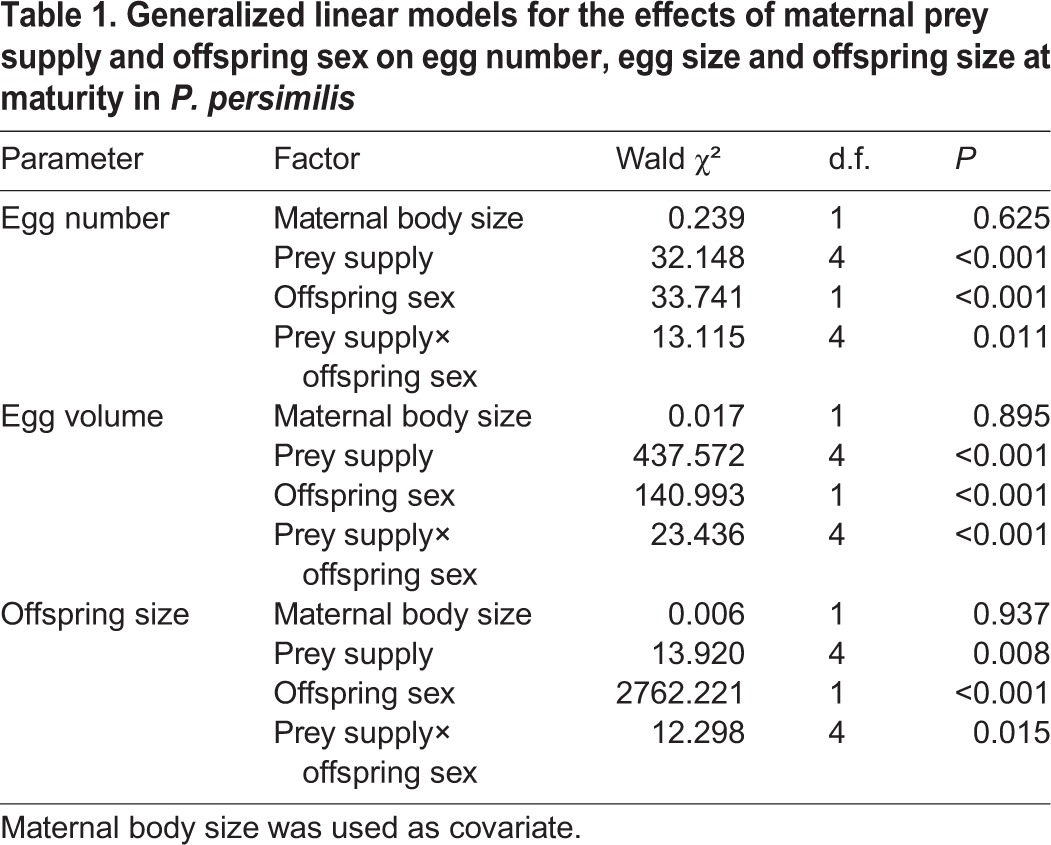
**Generalized linear models for the effects of maternal prey supply and offspring sex on egg number, egg size and offspring size at maturity in** ***P. persimilis***

**Table 2.**
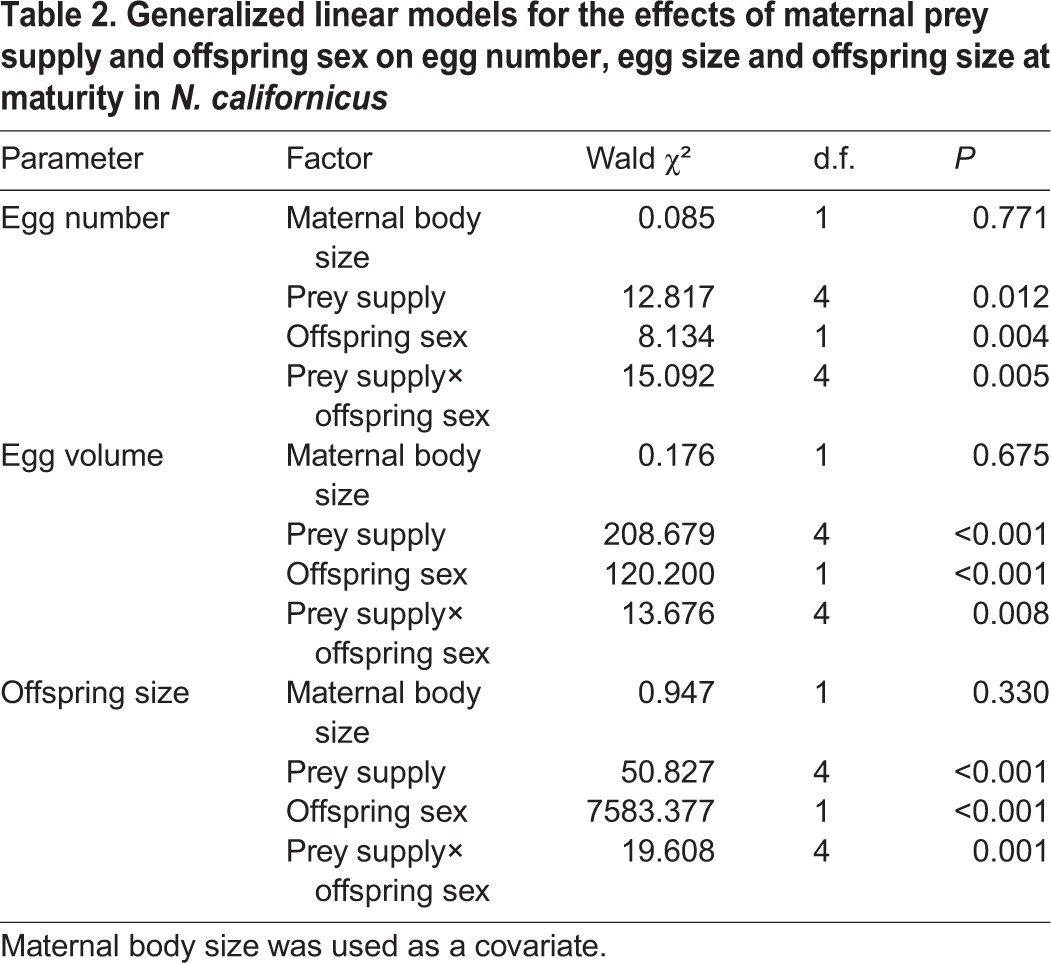
**Generalized linear models for the effects of maternal prey supply and offspring sex on egg number, egg size and offspring size at maturity in** ***N. californicus***

**Table 3.**
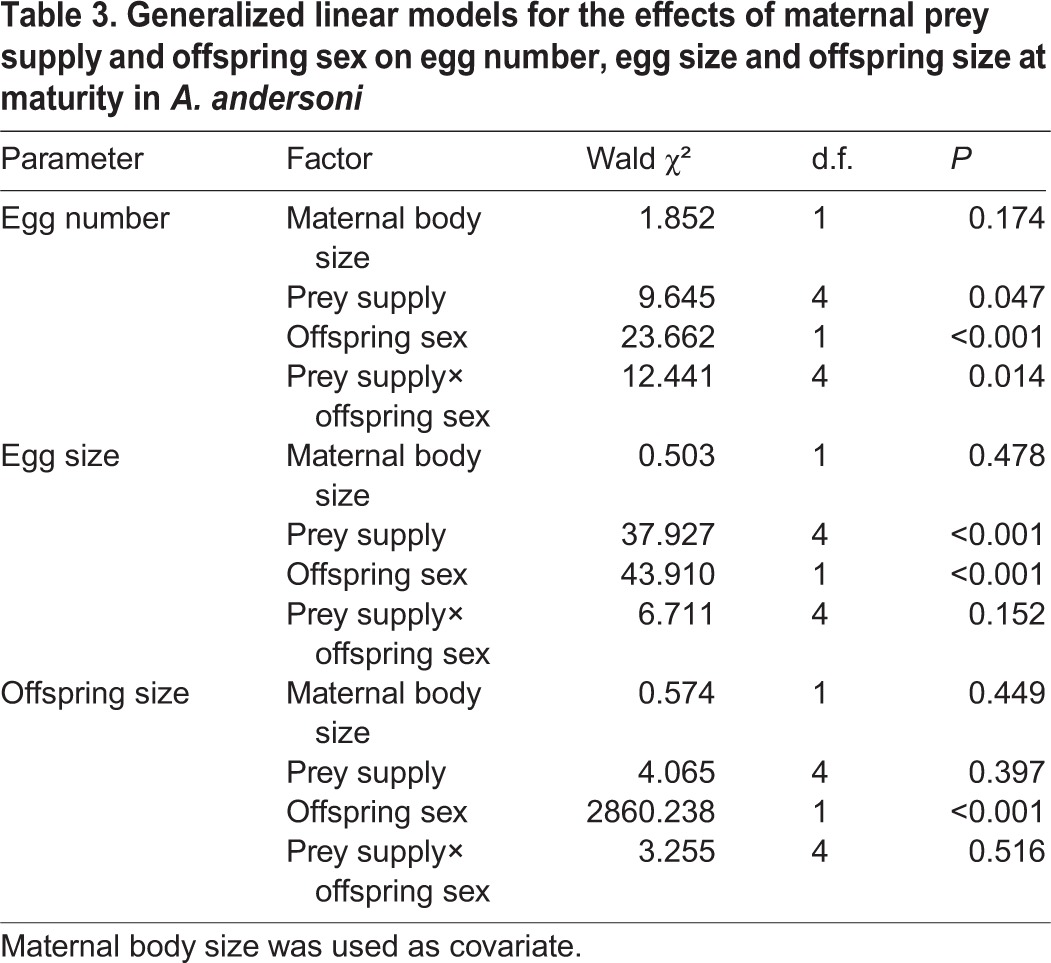
**Generalized linear models for the effects of maternal prey supply and offspring sex on egg number, egg size and offspring size at maturity in** ***A. andersoni***

### Sex-specific egg size

In each species, the egg sizes were independent of maternal body size but were affected by prey density. Additionally, offspring sex influenced egg sizes. The interaction term was only significant in *P. persimilis* and *N. californicus* but not *A. andersoni* (Tables 1–3). In *P. persimilis*, male eggs were always smaller than female eggs (*P*<0.005 for all pairwise comparisons at each prey density). Both male and female eggs were smaller at 9 spider mites than at higher prey densities (*P*<0.01 for all pairwise comparisons). Female and male egg sizes peaked at prey densities of 15 and 30 spider mites, respectively. Higher prey densities did not affect male egg size, whereas female egg size decreased (15 versus 45 and 75 spider mites, 30 versus 45 and 75: *P*<0.01) (Fig. 2B). Male and female eggs of *N. californicus* were similarly sized at 9 spider mites (*P*=0.223), and were smaller than those produced at higher prey densities (*P*<0.01 for all pairwise comparisons). Both male and female egg sizes were unaffected by prey densities of 15 or more spider mites, but female eggs were larger than male eggs (male versus female eggs at 15, 30, 45 and 75 spider mites: *P*<0.001) (Fig. 2E). Independent of offspring sex, eggs of *A. andersoni* were the smallest at 9 spider mites (*P*<0.001 for all pairwise comparisons) and similarly sized at prey densities of 15 or more spider mites. Across prey densities, male eggs (0.020±0.00012 µl; mean±s.e.m.) were smaller than female eggs (0.021±0.00011) (Fig. 2H).

### Trade-off between egg size and number

**Fig. 3.**
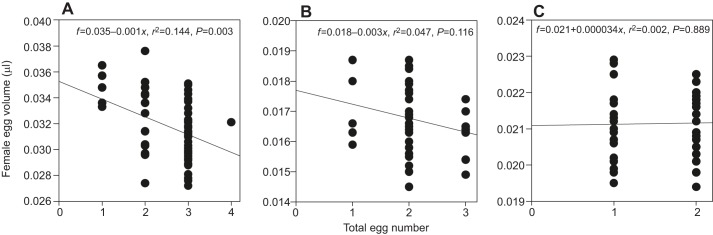
**Trade-off between egg number and size.** Female egg volumes of *P. persimilis* (A), *N. californicus* (B) and *A. andersoni* (C) as a function of egg number. Regression statistics and significance values are shown.

At prey densities of 9 spider mites, most females, produced only a single, small egg (if any), excluding the possibility of trading off egg size and number. Thus, correlation analyses between egg size and number were only conducted for female eggs at prey densities of 15 or more spider mites (the number of male eggs was not affected by prey density). Egg number was negatively correlated with female egg size in *P. persimilis*. A similar but non-significant trend was observed in *N. californicus*, but not in *A. andersoni* (Fig. 3).

### Sex-specific offspring body size at maturity

In no species did maternal body size affect offspring body size at maturity (Tables 1–3). Body size of *P. persimilis* offspring was affected by prey density, offspring sex and their interaction (Table 1). Prey density did not influence the body size of sons, whereas the body size of daughters was the smallest at 9 spider mites (*P*<0.01 for all pairwise comparisons). Daughters from mothers provided with 15 spider mites were larger than daughters from mothers provided with 45 (*P*=0.032) and 75 spider mites (*P*=0.016) (Fig. 2C). The body size of *N. californicus* offspring was influenced by prey density, offspring sex and their interaction (Table 2). The body size of sons was unaffected by prey density, whereas daughters grew smaller when their mothers were only provided with 9 spider mites (*P*<0.01 for all pairwise comparisons) (Fig. 2F). The body size of *A. andersoni* was affected only by offspring sex, not prey density and the interaction (Table 3): sons were smaller than daughters across prey densities (Fig. 2I).

## DISCUSSION

The level of adaptation to maternal food stress and the complexity of maternal response corresponded with the degree of specialization on spider mite prey in the three predator species. Most *A. andersoni* females, which are poorly adapted to exploit spider mite prey, did not oviposit at severe maternal food stress (9 spider mites), whereas the females of *N. californicus* (generalist predator with a preference for spider mites) and *P. persimilis* (specialized predator of spider mites) did oviposit. As expected, maternal prey supply did not influence the number of male eggs, whereas the number of female eggs increased with maternal prey supply resulting in a shift of offspring sex ratio from equally balanced (severe maternal food stress) to strongly female biased (optimal maternal food supply). Irrespective of species and offspring sex, the mothers reduced egg size at severe maternal food stress but maternal effects on offspring body size at maturity manifested only in *N. californicus* and *P. persimilis*. In both species, small egg size correlated with the body size of daughters but not sons. An additional sex-specific maternal effect was observed in *P. persimilis*: at moderate food stress (15 spider mites), the females produced the largest female eggs, giving rise to the largest daughters.

### Species-specific oviposition decisions under maternal food stress

In general, females experiencing local spider mite limitation may adopt two basic behavioral strategies. First, the females could postpone oviposition, search for adequate prey patches and, after recovering from starvation, resume egg production (Amano and Chant, 1978; Gotoh and Tsuchiya, 2009). Second, the females could stay in the prey patch and adjust egg number and size to the local food conditions (Vanas et al., 2006). The fact that most *A. andersoni* females did not oviposit at 9 spider mite eggs reflects the low nutritional value of spider mite prey for this predator, allowing only survival, but not reproduction under severe food stress. Additionally, juvenile *A. andersoni* are poorly adapted to survive at low spider mite densities (Walzer and Schausberger, 2011a). Thus, postponing oviposition and searching for richer prey patches is an adequate strategy for *A. andersoni* females, enhancing their own and offspring fitness.

In contrast to *A. andersoni*, most *P. persimilis* and *N. californicus* females produced some eggs under severe food stress. We propose three inter-related reasons to explain the oviposition decisions of *P. persimilis*, which is specialized on spider mite prey, and *N. californicus*, a generalist preferring spider mite prey. First, for both species, spider mites are high-quality food, allowing maximum egg production at surplus prey. For *P. persimilis*, spider mites are the only known food resource allowing egg production (McMurtry and Croft, 1997). Second, spider mites constitute an ephemeral and patchily distributed food, unpredictably varying in quality and quantity over space and time (Sabelis and Dicke, 1985). Thus, dispersal and searching for new spider mite prey patches should have higher costs for the females in terms of survival and fecundity than staying in the prey patch and adjusting egg production to the local food conditions. Third, juveniles of both species have relatively good survival probabilities at severe food stress because of being able to accelerate development under these conditions (Walzer and Schausberger, 2011a).

### Influence of maternal food stress on sex-specific egg number and size

Maternally controlled offspring sex ratio of phytoseiid mites is female-biased under optimal environmental conditions (Sabelis et al., 2002). Environmental cues decreasing the proportion of female eggs are a high density of conspecific females (Nagelkerke and Sabelis, 1998), small male mate body size (Walzer and Schausberger, 2014) and low maternal food supply (Amano and Chant, 1978; Momen, 1996; Toyoshima and Amano, 1998). In our study and in all three species, severe maternal food stress resulted in fewer female but not male eggs and a balanced sex ratio. The production costs are higher for large female than small male eggs (Toyoshima and Amano, 1998; Sabelis et al., 2002), thus, decreasing female rather than male egg production saves more energy for the mother's own survival. Ultimately, food-stressed females may also gain in fitness by shifting the sex ratio in favor of the less susceptible sex. For example, parrot finches produce more sons under food stress because sons are more likely to survive with limited food than are daughters (Pryke and Rollins, 2012). Similarly, in phytoseiid mites, daughters need more prey for development than the smaller and more quickly developing sons (Walzer and Schausberger, 2011a) and are thus more susceptible to food stress. Egg size, a pivotal life history trait, commonly correlates with other fitness components of juvenile development such as hatching success and survival (reviewed by Roff, 1992). Consequently, selection should favor females, which increase the investment per offspring, i.e. larger egg size, at the expense of egg number under food stress. In our experiments, however, mothers of all three species produced the smallest male and female eggs under the most severe food stress conditions. The most parsimonious explanation is that the available nutrients only allowed the production of a single, small egg, rendering trade-off of egg size and number impossible. Moderate food stress allowed the production of more than one egg, and *P. persimilis*, but not *N. californicus* and *A. andersoni*, increased its investment per offspring at the expense of egg number and produced few but disproportionally large female eggs. At surplus prey density, female egg size of *P. persimilis* decreased again at the expense of increased egg number. Trade-offs between egg number and size are typical for many vertebrates and arthropods (Bernardo, 1996; Fox and Czesak, 2000), but sex-specific trade-offs, as observed in *P. persimilis*, have rarely been documented (Saeki et al., 2009). This sex-specific trade-off was also weakly apparent in *N. californicus*, but was not present at all in *A. andersoni*. Uncontrolled variations in maternal age and size are commonly named methodological flaws that obscure the egg number/size trade-off (Fox and Czesak, 2000; Roff, 2001). However, in our experiments, maternal age was standardized and maternal body size did not influence egg size and number. The weak trade-off in *N. californicus* may be due to an insufficient number of replicates compromising statistical power. The lack of a trade-off in *A. andersoni* may be due to spider mites being a suboptimal prey; this species only partly sucks out spider mite eggs (Walzer and Schausberger, 2011a) and has higher oviposition rates with other food types (Duso and Camporese, 1991).

### Sex-specific maternal effects on offspring body size at maturity

In *N. californicus* and *P. persimilis*, severe maternal food stress only affected daughters, not sons: the females produced small male and female eggs and their daughters, but not sons, were also smaller, although they were reared under resource-rich environmental conditions. These sex-specific maternal effects follow the common trend in sexually size-dimorphic arthropods whereby food stress during reproduction more strongly affects the larger offspring sex (Fox and Czesak, 2000; Walzer and Schausberger, 2013). Additionally, in both species, male body size is less plastic than female body size, indicating the higher costs of being small in males than in females (Walzer and Schausberger, 2011a). This is especially evident in *P. persimilis* (Walzer and Schausberger, 2014, 2015). Maternal effects are generally considered adaptive if the offspring environment matches the maternal environment (Mousseau and Fox, 1998). Thus, if offspring suffer themselves from severe food limitation, being small may be advantageous because small female juveniles need less prey for development than their larger counterparts. Additionally, small females producing small eggs need less energy for egg production compared with large females, resulting in higher reproductive success (Spence et al., 1996). Conversely, under moderate food stress, only *P. persimilis* mothers produced the largest female eggs, from which the largest daughters also hatched. Moderate food stress allowed increased egg production, which should intensify food competition among hatching offspring. Under these circumstances, *P. persimilis* females apparently favor larger female offspring emerging from larger eggs because of their superior competitive abilities (Fox and Czesak, 2000).

## MATERIALS AND METHODS

### Species origin and rearing

*Phytoseiulus persimilis* Athias-Henriot 1957, *Neoseiulus californicus* (McGregor 1954) and *Amblyseius andersoni* (Chant 1957) used in experiments derived from laboratory-reared populations founded with specimens sampled in Sicily (Walzer and Schausberger, 2011a). Rearing units consisted of plastic tiles resting on water-saturated foam cubes in plastic boxes half-filled with water. The predators were fed at 2 to 3 day intervals with the spider mite *Tetranychus urticae* (Acari: Tetranychidae) by adding spider-mite-infested bean leaves (*P. persimilis, N. californicus*) or by brushing spider mites from infested leaves (*A. andersoni*) onto arenas (Walzer and Schausberger, 2011a).

To exclude inadvertent effects of maternal age on egg number and size (Fox and Czesak, 2000), we used cohorts of the same-aged females. To this end, eggs of *A. andersoni*, *N. californicus* and *P. persimilis* were randomly withdrawn from the rearing units and transferred to detached, spider-mite-infested bean leaves placed upside down on water-saturated foam cubes in plastic boxes (20×20×6 cm). After 8 days the predators had reached adulthood and were mated. Mated females were singly transferred into closed acrylic cages and starved for 24 h. Each cage consisted of a cylindrical cell (15 mm in diameter and 3 mm high) with a fine mesh screen at the bottom and closed on the upper side with a microscope slide (Schausberger, 1997). Only females producing at least one egg during the starvation period were used for experiments.

### Experimental units

Each experimental unit consisted of a circular disc (3.5 cm diameter), punched out from a trifoliate bean leaf, placed upside down on an agar cylinder (3.5 cm diameter, 3.5 cm high) in a plastic box (5×5×3.5 cm) and filled with water up to the leaf margin to confine the mites to the leaf disc. To obtain a given spider mite density, 1 to 5 ovipositing spider mite females were placed on each experimental unit for 24 h. After removing the spider mite females, the number of their eggs was adjusted to predetermined densities (6, 10, 20, 30 and 50 eggs). Immediately before the start of the experiment, spider mite larvae were added onto leaf discs (3, 5, 10, 15 and 25 larvae) to reach a 2:1 egg:larva ratio. A small plastic square (0.7 cm) served as shelter and oviposition site for the predator females on the leaf discs. Rearing and experimental units were kept in a climate chamber at 25±1°C, 70±5% RH and 16 h:8 h light:dark photoperiod.

### Effects of maternal food stress on sex-specific egg number and size, and offspring size at maturity

Single gravid females of *A. andersoni* (*N*=16–34 replicates per prey density), *N. californicus* (*N*=16–19) or *P. persimilis* (*N*=12–19) were placed on spider-mite-infested bean leaf discs for 24 h. After removing the predator females, the deposited eggs (*A. andersoni* eggs: *N*=14–31 per prey density; *N. californicus* eggs: *N*=22–35; *P. persimilis* eggs: *N*=14–51) were counted and egg widths (a) and lengths (b) non-destructively measured under the microscope at 200× magnification. The egg volumes were calculated using the formula for a prolate spheroid:
(1)
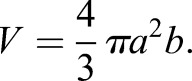



After size measurements, the eggs were placed singly on detached bean leaves and provided with surplus spider mite prey until they reached adulthood. After determining offspring sex, based on body size characteristics, both the females and their adult offspring were mounted in a drop of Hoyer's medium on microscope slides (Krantz and Walter, 2009). After drying the slides at room temperature for 2 days, the dorsal shield lengths of the mothers and their adult offspring were measured under the microscope at 200× magnification. Dorsal shield length is an appropriate indicator of species-specific body size of phytoseiid mites (Croft et al., 1999; Walzer and Schausberger, 2011a). Since offspring sex could only be determined for individuals reaching adulthood, the offspring sex ratios correspond to secondary sex ratios.

### Statistical analysis

SPSS 18.0.1 (SPSS Inc., 2006) was used for all statistical analyses. Generalized linear models (GLMs) were used to analyze the effects of species on the oviposition probability of the females (proportion of ovipositing females, binomial distribution, logit link function) at prey densities of 9 and 15 spider mites separately, because all females of all three species produced at least one egg at prey densities of 30 or more spider mites (Fig. 1A). The effects of species, maternal prey supply and their interaction on oviposition (number of deposited eggs, normal distribution and identity link function) was also analyzed by using GLMs. Additionally, the effects of maternal prey supply and offspring sex on egg number, egg volume and offspring body size at maturity (normal distribution, identity link function) were analyzed within each species. Maternal body size was used as covariate in all analyses. Sex-specific mean of egg sizes and offspring body sizes per mother were calculated when mothers produced more than a single female or male egg. To detail differences between maternal prey supply levels within and between species or within and between the offspring sexes, pairs of the estimated marginal means were compared by Fisher's least significant difference (LSD) tests if needed. Finally, the correlation of egg size and number was analyzed for each species separately by linear regression to detect potential trade-offs between these two pivotal life history traits.
